# Incidental finding of esophageal eosinophilia in patients investigated for suspected celiac disease: results from a pediatric retrospective study

**DOI:** 10.1007/s00431-026-07226-5

**Published:** 2026-07-21

**Authors:** Roberta Mandile, Carla Ziello, Erasmo Miele, Riccardo Troncone, Renata Auricchio

**Affiliations:** 1 Department of Translational Medical Sciences, University Federico II of Naples, Naples, Italy; 2 European Laboratory for Food Induced Disease (ELFID), University Federico II of Naples, Naples, Italy

**Keywords:** Celiac disease, Esophageal eosinophilia, Eosinophilic esophagitis

## Abstract

Incidental esophageal eosinophilia (EE) can be detected in children investigated for suspected celiac disease (CD), but the relationship between CD and eosinophilic esophagitis (EoE) remains unclear. This study describes the characteristics of these patients and evaluates whether EE resolves on a gluten-free diet (GFD). This retrospective study included all patients < 18 years biopsied for suspected CD (2018–2025). When esophageal abnormalities were observed, additional biopsies were taken. Cases (CD + EE) had positive antitransglutaminase antibodies—classified as active CD (ACD) or potential CD (PCD) if presented or not intestinal damage- and biopsy-proven EE (≥ 15 eos/HPF). Comparators were randomly selected among CD-diagnosed patients without macroscopical esophageal abnormalities (CD + EE −). Clinical, immunological, and histological features were compared. EE remission (< 15 eos/HPF) was assessed in cases after treatment. PCD patients received proton pump inhibitors (PPIs) for 8 weeks, with GFD prescribed only if PPIs failed. ACD patients received a 6-month GFD first, with PPIs added if esophageal remission was not achieved. 14/520 children (2.7%) had confirmed EE (9 ACD, 5 PCD). Only 2/14 (14%) were symptomatic. Compared to 100 CD + EE − subjects, cases were significantly more likely to be male (86% vs. 34%; *p* = 0.0003), atopic (57% vs. 22%; *p* = 0.009), and having peripheral eosinophilia (86% vs. 12%; *p* < 0.0001). Overall, GFD induced EE remission in 5/11 (45%) of cases. Most ACD patients required additional PPI therapy after GFD failure.

* Conclusion:*  Incidental EE affects ~ 3% of pediatric CD evaluations. These patients typically fulfill histological EoE criteria and are often asymptomatic. Male sex, atopy, and peripheral eosinophilia are strongly associated to this condition. While GFD resolves EE in nearly half of cases, the persistence in others suggests EE is frequently an independent comorbidity. Nonetheless, a GFD is a viable first-line approach in dual-diagnosis patients.

**Table:** 

**What is Known:** • *Incidental esophageal eosinophilia (EE) is sometimes found in children being evaluated for celiac disease (CD)*. • *The precise clinical relationship and overlap between celiac disease and eosinophilic esophagitis (EoE) remain unclear*.
**What is New:** • *Incidental EE affects ~3% of pediatric CD cases; patients are often asymptomatic but strongly linked to male sex, atopy, and peripheral eosinophilia*. • *A gluten-free diet solves EE in aroung 45% of cases, making it a viable first-line approach in dual diagnosis of EE and celiac disease, though others additional, EE specific therapies*.

**What is Known:**

• *Incidental esophageal eosinophilia (EE) is sometimes found in children being evaluated for celiac disease (CD)*.

• *The precise clinical relationship and overlap between celiac disease and eosinophilic esophagitis (EoE) remain unclear*.

**What is New:**

• *Incidental EE affects ~3% of pediatric CD cases; patients are often asymptomatic but strongly linked to male sex, atopy, and peripheral eosinophilia*.

• *A gluten-free diet solves EE in aroung 45% of cases, making it a viable first-line approach in dual diagnosis of EE and celiac disease, though others additional, EE specific therapies*.

## Introduction

Celiac disease (CD) and eosinophilic esophagitis (EoE) are two immune-mediated gastrointestinal disorders that predominantly affect the pediatric population with a prevalence of respectively in 1–2% [1] and 0.05% [2]. Celiac disease is triggered by a Th1 immune reaction to gluten, leading to inflammation of the intestinal mucosa and subsequent villous atrophy [3]. Clinical manifestations are highly variable, ranging from classic gastrointestinal symptoms to extraintestinal signs and even completely asymptomatic forms [4]. Regardless of symptomatology, the presence of clear enteropathy requires the initiation of a strict and lifelong gluten-free diet (GFD) [5]. Conversely, in cases of positive celiac disease autoantibodies but architecturally normal duodenal mucosa (a condition known as potential celiac disease), a gluten-free diet is not necessarily recommended unless the patient experiences disabling symptoms [6, 7].

Eosinophilic esophagitis (EoE) is characterized by eosinophil-predominant inflammation of the esophageal mucosa (> 15 eosinophils per high-power field, along with other specific histological alterations) generally accompanied by esophageal dysfunction symptoms. In 4 to 23% of the cases, endoscopy in EoE can be macroscopically normal, but more often it appears altered, with a variable combination of exudate, rings, furrows, and strictures [8]. Clinical presentation, as in celiac disease, can be extremely variable and is strongly influenced by the patient’s age. In infants and young children, symptoms often mimic those of gastroesophageal reflux disease, whereas with increasing age, symptoms more clearly reflect esophageal dysfunction. The most characteristic symptom is dysphagia, which may progress to food impaction. Treatment for EoE includes high-dose proton pump inhibitors (PPIs), topical corticosteroids, and elimination diets [9]. However, no universally accepted first-line therapy has yet been established. Biologics like dupilumab can also be considered in case of first-line treatment failure. Each treatment option should be tailored to the individual patient based on personal risk factors. Although EoE is a relatively newly recognized disease, data from adult populations suggest a 5–10% annual risk of progression to fibrostenotic forms in cases of inadequate treatment response (defined as esophageal eosinophil counts remaining above 15/HPF) [10]. Likewise, despite the scarcity of long-term safety and efficacy data, particularly in pediatric cohorts, current evidence suggests that maintenance therapy at the lowest effective dose should be continued indefinitely to sustain remission and prevent complications [9]. Even more complex is the case of patients in whom esophageal eosinophilia (EE) is not accompanied by characteristic symptoms and thus appears as an incidental finding. This is the situation, for example, of patients in whom EE is detected incidentally during an endoscopic examination performed for an unrelated diagnostic suspicion, such as celiac disease.

Since several prospective and retrospective studies have reported a higher-than-expected co-occurrence of EoE and CD [11–15], it is hard to say whether the incidental finding of EE in patients investigated for suspected CD is consistent with an EoE diagnosis or represents a different condition, possibly related to celiac disease itself and so responsive to a gluten-free diet (GFD).

In the present study, we aim to clinically, endoscopically, and histologically characterize a cohort of pediatric patients investigated for suspected celiac disease (positive IgA antitransglutaminase antibodies) in whom concomitant esophageal eosinophilia is incidentally diagnosed. Secondly, we aim to evaluate whether esophageal eosinophilia in CD patients is reversible after GFD or represents independent, coexisting EoE condition.

## Methods

### Study design

We retrospectively recruited all pediatric patients (< 18 years) referred to our center for suspected celiac disease (defined by positive antitransglutaminase antibodies of IgA class at any titer in two separate determinations, confirmed by antiendomysial antibodies) biopsied from January 2018 to December 2025.

Among them, we selected patients presenting with endoscopic abnormalities of the esophagus suggestive of eosinophilic esophagitis represented by fissures, strictures, rings, and esophageal stenosis, with or without calculation of the EoE endoscopic reference score (EREFS score). All patients meeting these inclusion criteria underwent, in addition to the duodenal biopsies required for the diagnosis of celiac disease, additional esophageal biopsies: two from the upper segment of esophagus, two from the middle, and two from the lower.

In cases where histological analysis confirmed esophageal eosinophilia (> 15 eosinophils per high-power field), a specific treatment was proposed—dietary (gluten-free diet, GFD) or proton pump inhibitors (PPIs)—and esophageal eosinophilia was reassessed via a follow-up upper endoscopy (EGDS) scheduled after 8–12 weeks if pharmacological treatment was prescribed, or 6 months if GFD alone was prescribed. Adherence to GFD was assessed by a nutritional interview performed by an expert dietitian, together with the examination of intestinal architecture when the biopsy was re-performed for EE monitoring.

As comparator group, we performed a computer-assisted simple random sampling to select patients who underwent an EGDS for suspected and then diagnosed celiac disease during the same period, in a number equal to at least twice the number of cases. Patients with IgA deficiency were excluded.

Patients of both groups (case group: CD + EE +; comparator group: CD + EE −) were analyzed for their main clinical, epidemiological, and histologic features. Histological response of esophageal eosinophilia to gluten free diet (when prescribed) was recorded. *Responders to GFD* were defined as patients in whom esophageal eosinophilia was reduced to less than 15 eosinophils/HPF in all the three esophageal segments. Patients gave their informed consent and the study was approved by the University Federico II ethical committee.

### Clinical and laboratory variables

For each patient, we collected clinical data like age, sex, age at diagnosis, family history of CD, atopic predisposition (defined as personal and/or family history of allergic disease and/or objective evidence of IgE-mediated sensitization), upper and lower gastrointestinal symptoms, biochemical data like peripheral eosinophilia (defined as an absolute count of eosinophils in peripheral blood higher than 500 cells/mcl) and histological data both from the intestinal mucosa (Marsh grade, villi to crypt ratio and number of eosinophils/HPF), and the esophageal mucosa (number of eosinophils/HPF; 1 HPF = 0.24 mm^2^).

### Antibody measurement

To measure serum antitransglutaminase (anti-TG2) antibodies, an enzyme-linked immunosorbent assay kit was used, based on a human recombinant antigen (Eu-tTg IgA; Eurospital, Trieste, Italy). The cutoff point for positivity was 9 IU. Serum IgA EMA was measured by indirect immunofluorescence using 7-mm-thick frozen sections of human umbilical cord as the source of antigen. The samples were considered positive if a thin fluorescent network appeared around the smooth muscle fibers. Minimum positive dilution was 1:10.

### Esophageal and duodenal biopsies and histological analysis

In each patient, esophagogastroduodenoscopy with 5 biopsies, 1 from the bulb and 4 from the distal duodenum, was carried out. According to our protocol [16], 4 of 5 fragments, including 1 from the bulb, were fixed in 10% formalin, embedded in paraffin, and then stained with hematoxylin. Histological and morphometrical analyses by light microscopy were performed by 2 experienced pathologists. A villous height: crypt depth (v/c) ratio 2 was considered normal, while patients with a v/c ratio < 2 had a clear picture of enteropathy and were thus classified as celiac. Among biopsies with a normal villous height: crypt depth ratio, Marsh 0 was defined by the presence of less than 25 intraepithelial lymphocytes (IELs) per 100 enterocytes and Marsh 1 by the presence of more than 25 IELs per 100 enterocytes. The Marsh score was given based on the score of the fragment with the worst picture.

In patients who exhibited macroscopical alterations suggestive of EoE (esophageal rings—trachealization, linear furrows, white exudates or plaques, edema, mucosal pallor, strictures, and narrow-caliber esophagus) additional esophageal biopsies were obtained: at least 2 from the proximal, 2 from the medium, and 2 from the distal esophagus. Histological confirmation of EoE was defined by the presence of at least 15 eosinophils/HPF in at least one of the segments explored [9].

### Statistical analysis

Statistical analysis was performed using IBM SPSS Statistics (IBM Corp., Armonk, NY, USA). Categorical variables were expressed as percentages and compared using the *χ*^2^ test or Fisher’s exact test, as appropriate. Continuous variables were reported as mean and range and compared using Student’s *t*-test or the Mann–Whitney *U* test according to data distribution. Odds ratios (OR) with 95% confidence intervals (CI) were calculated to identify factors associated with esophageal eosinophilia. A two-tailed *p* value < 0.05 was considered statistically significant.

### Ethical considerations

The study was conducted in accordance with the Declaration of Helsinki. Ethical approval was obtained from the institutional review board. As this was a retrospective analysis of anonymized data, informed consent was waived.

## Results

### Clinical and histological features of patients investigated for CD with incidental finding of EE compared to patients without

Five hundred twenty patients were biopsied between January 2018 and November 2025 in suspicion of CD for anti-TG2 positivity at any level, in at least 2 separate blood samples. Among them, 18 presented macroscopical alterations of the esophagus suggestive for EoE. All of them received additional esophageal biopsies and in 14/18 (study group) the histological evaluation confirmed the presence of an increased number of eosinophils in least one of the esophageal segments (> 15 eosinophils/HPF). All patients were off therapy and with no dietary restriction at the time the EGDS was performed. The mid esophageal segment showed the highest degree of eosinophilic infiltration, with counts ranging from 12 to 65 eosinophils per high-power field, representing the most extensively involved region among those examined, despite differences among the segments were not statistically significant. In 9/14 cases (64%), the duodenal mucosa appeared to be damaged (villi to crypt ratio < 2) and a diagnosis of classical form of CD (ACD) was posed. In 5/14 cases (35%) the positive CD associated serology was not coupled by duodenal damage and a potential celiac disease (PCD) diagnosis was posed.

To identify a possible risk factor associated to the finding of concomitant esophageal eosinophilia in patients investigated for suspected CD, we selected a comparator group of patients that received a duodenal biopsy in the same period of time for suspected CD (anti-TG2 positive at any level in at least two separate blood samples) but did not show macroscopical esophageal alterations suggestive for EoE. 100 patients were thus randomly recruited from our celiac database (66 ACD—34 PCD).

Patients with concomitant EE (cases) were more frequently male (12/14 patients, 86%), mean age at diagnosis was 6.7 years (range 2–13), family history of CD was reported in 3/14 (21%) of the cases. A positive history of atopy, defined as the presence of personal and/or familiar history of allergy and/or objective signs of IgE mediated sensitization (positive results on skin prick tests (SPT), specific IgE blood tests, or elevated total IgE, tested in case of suggestive clinical manifestation), was present in 8/14 (57%) cases. The 85% of patients (12/14) presented peripheral eosinophilia (mean 760, range 550–1550 cells/µL). Symptoms reported were mainly lower gastrointestinal symptoms. In only two cases upper gastrointestinal symptoms were reported (gastroesophageal reflux and post prandial cough), but in only one of them (reflux) this was the primary reason that prompted the patient to have a medical consultation. Specular descriptive analysis was performed in the comparator group (Table 1).

**Table 1:** Case group: patients with CD + EE +; comparator group: patients with CD + EE +

Variable	Cases (*n* = 14)	Controls (*n* = 100)	OR (95% CI)*	*p*
Male sex	12/14 (86%)	34/100 (34%)	11.65	0.0003
Mean age at diagnosis	6.7 y.o (2–13)	7.2 y.o (1.2–17.5)		0.53
CD family history	3/14 (21%)	39/100 (39%)	0.43	0.25
Atopy	8/14 (57%)	22/100 (22%)	4.73	0.009
Peripheral eosinophilia	12/14 (86%)	12/100 (12%)	44.0	< 0.0001
Mean blood eosinophil count (cells/μL)	760.7 (370–1550)	277.4 (10–1670)		< 0.0001
Anti-Tg (ULN)	24.67 (1.70–165.52)	12.97 (1.03–165.52)	0.35	0.22
MARSH 3	9/14 (64%)	65/100 (65%)	0.97	1.00

*OR* odds ratio, *CI* confidence interval, *ULN* upper limit of normal

Main risk factors to identify an incremented number of esophageal eosinophils and thus to distinguish case group (CD + EE +) from comparator group (CD + EE −) were in order: presence of peripheral eosinophilia (86% vs 12%, OR 44, *p* < 0.0001), male sex (86% vs 34%; OR 11.65; *p* = 0.00031), atopy (57% vs 22%, OR 4.73; *p* = 0.009). No significant difference was observed in the prevalence of CD family history (21% vs 39%; OR 0.43; *p* = 0.25).

### Histological response of esophageal eosinophilia to GFD

**Fig. 1:**
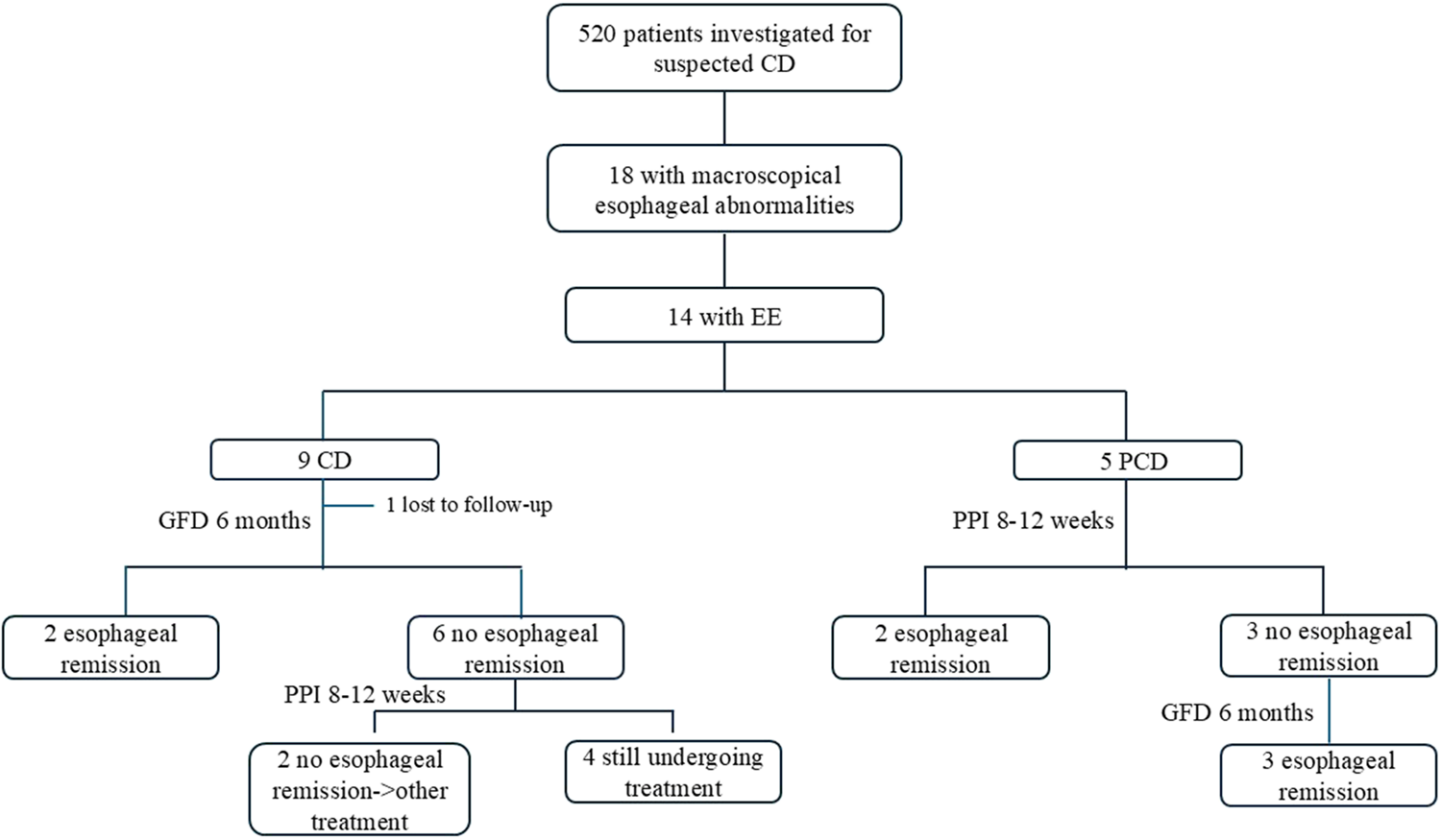
Management algorithm of patients enrolled in the study. EE: eosinophilic esophagitis, ACD: atrophic celiac disease, PCD: potential celiac disease

Among patients with confirmed celiac disease (CD, *n* = 9), all initiated GFD alone as first-line therapy for six months. One patient was lost to follow-up and was excluded from response evaluation. Of the remaining eight patients, two (2/8, 25%) achieved complete histological remission of esophageal eosinophilia and were classified as *responders to GFD*, while the other 6 did not reach complete histological remission (6/8, 75%) and were thus considered as *non-responder to GFD*. Of note 2/6 of these patients, even if did not reach complete histological remission, presented a clinically significant improvement (reduction from a mean value of 29 eos before GFD to 15 eos/HPF post GFD) and were thus continued on a GFD in the hypothesis a longer period of dietary treatment can induce complete remission (Fig. 1). All patients treated with a GFD experienced histologic remission of enteropathy as expected. In 6/8 patients where GFD failed in inducing esophageal remission, PPI were added for 8 weeks: 2 continued not to achieve remission, and 4 are still under treatment.

Among patients with potential celiac disease (PCD, *n* = 5), all received proton pump inhibitors (PPIs) as first-line therapy for 8 weeks. Two patients (2/5, 40%) achieved complete histological remission of esophageal eosinophilia after PPI treatment and were classified as responders to PPI. The remaining three non-responders to PPI were subsequently switched to a gluten-free diet (GFD), and all of them (3/3, 100%) achieved histological remission at follow-up endoscopy (*responders to GFD*).

Combining CD and PCD patients into a single cohort, 11 individuals were evaluable for histological remission. Overall, 5 of these 11 patients achieved complete histological remission with gluten-free diet alone, resulting in an overall remission rate of 45%

## Discussion

The incidental detection of esophageal eosinophilia in children undergoing upper endoscopy for suspected celiac disease represents a clinically relevant but still insufficiently characterized condition.

In our cohort, approximately 3% of children investigated for suspected CD were found to have histologically confirmed esophageal eosinophilia, a prevalence higher than the expected one in the general pediatric population, but comparable to what is reported in previous pediatric series evaluating children undergoing endoscopy for immune-mediated gastrointestinal disorders [11–14]. Our data could even underestimate the phenomenon of EE in CD, since in our cohort esophageal samples were taken only in case of macroscopical esophageal abnormalities and not as routine practice during the endoscopic procedure. The issue of whether the documented EE is sufficient to diagnose a EoE in asymptomatic or mildly symptomatic patients investigated for suspected CD remains controversial [13, 17]. Several studies have reported an increased coexistence of the two conditions, particularly in pediatric populations, suggesting shared pathogenic mechanisms related to food antigens and mucosal immune activation. Conversely, other studies and a recent systematic review failed to confirm a true epidemiological association, proposing that the observed coexistence may reflect increased diagnostic scrutiny and biopsy sampling rather than a causal link [18, 19].

Our data suggest that children with incidental EE, identified during CD evaluation, displayed a clinical and immunologic profile strongly resembling the one of classic EoE patients: male sex, atopic predisposition and peripheral eosinophilia were significantly more frequently reported in patients with EE compared to those without, with an OR of, respectively, 11, 4, and 44. Most of the patients did not report symptoms suggestive of esophageal dysfunction. In only one patient, regurgitation was reported, but it is actually not possible to say whether it was attributable to EoE or CD. Moreover, only less than half of the patients responded to GFD in terms of histological remission of EE (3/3 PCD patients and 2/8 ACD patients). This supports the idea that EE in CD actually configures a picture of associated asymptomatic EoE rather than a simple manifestation of CD. This is also in line with the different immunological mechanisms that sustain the two conditions: Th1 mediated in CD and Th2 in EoE.

Unfortunately, clinical significance and optimal management of asymptomatic EoE, with or without concomitant CD, remain uncertain, particularly in children. While untreated EoE has been associated with progressive fibrostenotic remodeling in adults [10, 20], pediatric data, especially in asymptomatic patients, are limited. Current guidelines generally recommend treating EoE with high doses of PPI, topical corticosteroids or dietary restrictions as first-line therapies to achieve histological remission, but these recommendations are largely based on symptomatic populations.

Our findings underscore the need for a more individualized approach when EE is detected incidentally in children evaluated for suspected CD.

A key finding of our study is that EE resolved with gluten-free diet alone in a minority of patients. Overall, less than half of evaluable patients achieved complete esophageal histological remission on GFD, indicating that gluten withdrawal may reduce eosinophilic inflammation in only selected cases, as reported in the few studies published in literature on the same issue [17, 21].

From a clinical perspective, a gluten-free diet represents a reasonable and low-risk first-line approach in asymptomatic or mildly symptomatic children with incidental EE identified during CD workup, as it may obviate the need for additional treatment in selected cases. Besides, gluten is one of the antigens implicated in the pathogenesis EoE itself, despite immunologic mechanisms different from those in CD [22]. However, persistence of esophageal eosinophilia should prompt the initiation of standard EoE-directed therapies in accordance with current guidelines [9, 23].

This study presents several limitations that should be considered. First, its retrospective nature and relatively small sample size warrant further validation through longitudinal, multicenter studies. Moreover, our data might underestimate both the prevalence of EE in CD and its response rate to a GFD. Indeed, esophageal biopsies were performed only in patients with macroscopical esophageal lesions. Furthermore, a GFD was not attempted in all PCD patients, as two individuals achieved remission with PPIs; nonetheless, we cannot exclude the possibility that a GFD might have also been effective.

## Conclusion

Incidental EE is diagnosed in approximately 3% of children evaluated for suspected CD. Male sex, atopy, and peripheral eosinophilia are strongly associated to its presence. While some patients improve on a GFD, most show persistent esophageal eosinophilia, indicating it represents usually an independent presentation of EoE rather than a CD-related manifestation. Still, a GFD may be considered a reasonable first-line approach and may obviate further treatment in asymptomatic children with both conditions.

## Data Availability

No datasets were generated or analysed during the current study.

## References

[1] Singh P, Arora A, Strand TA, Leffler DA, Catassi C, Green PH, Kelly CP, Ahuja V, Makharia GK (2018) Global prevalence of celiac disease: systematic review and meta-analysis. Clin Gastroenterol Hepatol 16:823-836.e2. 10.1016/j.cgh.2017.06.037 29551598 10.1016/j.cgh.2017.06.037

[2] Dellon ES, Jensen ET, Martin CF, Shaheen NJ, Kappelman MD (2014) Prevalence of eosinophilic esophagitis in the United States. Clin Gastroenterol Hepatol 12:589-596.e1. 10.1016/j.cgh.2013.09.008 24035773 10.1016/j.cgh.2013.09.008 PMC3952040

[3] Abadie V, Han AS, Jabri B, Sollid LM (2024) New insights on genes, gluten, and immunopathogenesis of celiac disease. Gastroenterology 167:4–22. 10.1053/j.gastro.2024.03.042 38670280 10.1053/j.gastro.2024.03.042 PMC11283582

[4] Husby S, Koletzko S, Korponay-Szabó I, Kurppa K, Mearin ML, Ribes-Koninckx C, Shamir R, Troncone R, Auricchio R, Castillejo G et al (2020) European society paediatric gastroenterology, hepatology and nutrition guidelines for diagnosing coeliac disease 2020. J Pediatr Gastroenterol Nutr 70:141–156. 10.1097/MPG.0000000000002497 31568151 10.1097/MPG.0000000000002497

[5] Luque V, Crespo-Escobar P, Hård Af Segerstad EM, Koltai T, Norsa L, Roman E, Vreugdenhil A, Fueyo-Díaz R, Ribes-Koninckx C (2024) Gluten-free diet for pediatric patients with coeliac disease: a position paper from the ESPGHAN Gastroenterology Committee, Special Interest Group in Coeliac Disease. J Pediatr Gastroenterol Nutr. 10.1002/jpn3.12079 10.1002/jpn3.12079 38291739

[6] Mandile R, Lerro F, Carpinelli M, D’Antonio L, Greco L, Troncone R, Auricchio R (2024) Potential celiac disease in children: health status on a long-term gluten-containing diet. Nutrients 16:1708. 10.3390/nu16111708 38892641 10.3390/nu16111708 PMC11174900

[7] Mandile R, Discepolo V, Scapaticci S, Vecchio M, Maglio M, Greco L, Troncone R, Auricchio R (2017) The effect of gluten free diet on clinical symptoms and the intestinal mucosa of patients with potential celiac disease. J Pediatr Gastroenterol Nutr 66:1. 10.1097/MPG.0000000000001745 10.1097/MPG.0000000000001745 28922261

[8] De Matteis A, Pagliaro G, Corleto VD, Pacchiarotti C, Di Giulio E, Villa MP, Parisi P, Vassallo F, Ziparo C, Di Nardo G (2020) Eosinophilic esophagitis in children: clinical findings and diagnostic approach. Curr Pediatr Rev 16:206–214. 10.2174/1573396315666191004110549 31584371 10.2174/1573396315666191004110549 PMC8193808

[9] Amil-Dias J, Oliva S, Papadopoulou A, Thomson M, Gutiérrez-Junquera C, Kalach N, Orel R, Auth MK-H, Nijenhuis-Hendriks D, Strisciuglio C et al (2024) Diagnosis and management of eosinophilic esophagitis in children: an update from the European Society for Paediatric Gastroenterology, Hepatology and Nutrition (ESPGHAN). J Pediatr Gastroenterol Nutr 79:394–437. 10.1002/jpn3.12188 38923067 10.1002/jpn3.12188

[10] Warners MJ, Oude Nijhuis RAB, de Wijkerslooth LRH, Smout AJPM, Bredenoord AJ (2018) The natural course of eosinophilic esophagitis and long-term consequences of undiagnosed disease in a large cohort. Am J Gastroenterol 113:836–844. 10.1038/s41395-018-0052-5 29700481 10.1038/s41395-018-0052-5

[11] Aly M, Liu BD, Song G (2024) Medical and demographic characteristics of patients with eosinophilic esophagitis and celiac disease: a retrospective cohort study. J Clin Gastroenterol. 10.1097/MCG.0000000000002105 39621387 10.1097/MCG.0000000000002105

[12] Kaval D, Akkuş E, Dogan G, Kiykim A, Cokugras H, Kepil N, Beser O, Cokugras F (2025) *Eosinophilic esophagitis *and food allergy in celiac disease. 10.21203/rs.3.rs-7482888/v1

[13] Cristofori F, D’Abramo FS, Rutigliano V, Dargenio VN, Castellaneta S, Piscitelli D, Benedittis DD, Indrio F, Raguseo LC, Barone M et al (2021) Esophageal eosinophilia and eosinophilic esophagitis in celiac children: a ten year prospective observational study. Nutrients. 10.3390/nu13113755 34836010 10.3390/nu13113755 PMC8625488

[14] Thompson JS, Lebwohl B, Reilly NR, Talley NJ, Bhagat G, Green PHR (2012) Increased incidence of eosinophilic esophagitis in children and adults with celiac disease. J Clin Gastroenterol 46:e6–e11. 10.1097/MCG.0b013e318221aefd 21778897 10.1097/MCG.0b013e318221aefd

[15] Bergman A, Greifer M, Levine J (2024) Concurrent celiac disease and eosinophilic esophagitis in a pediatric cohort: more than a coincidence. Clin Pediatr (Phila) 63:1573–1578. 10.1177/00099228241232876 38374667 10.1177/00099228241232876

[16] Maglio M, Marano A, Mandile R, Angelino P, D’Ambrosio A, Mirra A, Auricchio R, Greco L, Troncone R, Discepolo V (2025) Immunohistochemistry to highlight intraepithelial lymphocytes in formalin-fixed and frozen duodenal biopsies: comparability of results and cut-off revision. Dig Dis Sci. 10.1007/s10620-025-09168-8 40668482 10.1007/s10620-025-09168-8 PMC12531324

[17] Ari A, Morgenstern S, Chodick G, Matar M, Silbermintz A, Assa A, Mozer-Glassberg Y, Rinawi F, Nachmias-Friedler V, Shamir R et al (2017) Oesophageal eosinophilia in children with coeliac disease. Arch Dis Child 102:825–829. 10.1136/archdischild-2016-311944 28404554 10.1136/archdischild-2016-311944

[18] Hommeida S, Alsawas M, Murad MH, Katzka DA, Grothe RM, Absah I (2017) The association between celiac disease and eosinophilic esophagitis. J Pediatr Gastroenterol Nutr 65:58–63. 10.1097/MPG.0000000000001499 28045773 10.1097/MPG.0000000000001499

[19] Lucendo AJ, Arias Á, Tenias JM (2014) Systematic review: the association between eosinophilic oesophagitis and coeliac disease. Aliment Pharmacol Ther 40:422–434. 10.1111/apt.12859 25041372 10.1111/apt.12859

[20] Shaheen NJ, Mukkada V, Eichinger CS, Schofield H, Todorova L, Falk GW (2018) Natural history of eosinophilic esophagitis: a systematic review of epidemiology and disease course. Dis Esophagus 31:doy015. 10.1093/dote/doy015 29617744 10.1093/dote/doy015 PMC6102800

[21] Patton T, Chugh A, Padhye L, DeGeeter C, Guandalini S (2019) Pediatric celiac disease and eosinophilic esophagitis: outcome of dietary therapy. J Pediatr Gastroenterol Nutr 69:e43–e48. 10.1097/MPG.0000000000002343 30921260 10.1097/MPG.0000000000002343

[22] Mayerhofer C, Kavallar AM, Aldrian D, Lindner AK, Müller T, Vogel GF (2023) Efficacy of elimination diets in eosinophilic esophagitis: a systematic review and meta-analysis. Clin Gastroenterol Hepatol 21:2197-2210.e3. 10.1016/j.cgh.2023.01.019 36731591 10.1016/j.cgh.2023.01.019

[23] GIBoardReview.com (2020) ACG Clinical Guideline: diagnosis and management of eosinophilic esophagitis. The William M. Steinberg Board Review in Gastroenterology and Best Practices Course

